# Multifunctional activity of *Mentha piperita* essential oil nanoemulsion against *Helicobacter pylori*

**DOI:** 10.1038/s41598-026-52730-1

**Published:** 2026-05-18

**Authors:** Golnaz Ebrahimi, Bahareh Attaran, Ali Mohammadi

**Affiliations:** 1 Department of Microbiology, Faculty of Biological Sciences, Alzahra University, Tehran, Iran; 2 Research Center for Applied Microbiology and Microbial Biotechnology (CAMB), Alzahra University, Tehran, Iran

**Keywords:** *Helicobacter pylori*, Nanoemulsion, *Mentha piperita*, Antimicrobial activity, Anti-biofilm activity, Urease activity, Biotechnology, Drug discovery, Microbiology

## Abstract

*Helicobacter pylori* is a Gram-negative bacterium implicated in chronic gastric infections and gastric cancer, making it an important target for therapeutic intervention. Increasing antibiotic resistance of this pathogen has reduced the efficacy of conventional therapies, emphasizing the urgent need for alternative natural treatments. *Mentha piperita* essential oil exhibits antimicrobial and antioxidant activities, which are further enhanced when formulated as a nanoemulsion. Nanoemulsification improves solubility, stability, bioavailability, and bacterial targeting. Although essential oil–based nanoemulsions have shown antimicrobial potential, limited studies have systematically evaluated their effects on key virulence factors such as urease activity and biofilm formation in *H. pylori*. In this study, a *Mentha piperita* essential oil nanoemulsion stabilized with sodium dodecyl sulfate (MPNE–SDS) exhibited a mean particle size of 168 nm, a Polydispersity Index of 0.28, and maintained stability for up to three months. MPNE–SDS demonstrated potent antibacterial activity against *H. pylori*, with MIC values ranging from 6.25 to 50 µL/mL of nanoemulsion formulation, and showed enhanced efficacy compared to bulk essential oil. It also reduced whole-cell urease activity (IC₅₀ = 41.6 µL/mL) and inhibited biofilm formation (IC₅₀ = 24.5 µL/mL), while higher concentrations were required for biofilm eradication (EC₅₀ = 133 µL/mL). These findings indicate that MPNE–SDS enhances antibacterial performance and affects key *H. pylori* virulence-associated activities, suggesting its potential as a non-antibiotic approach and warranting further preclinical investigation.

**Supplementary Information:**

The online version contains supplementary material available at 10.1038/s41598-026-52730-1.

## Introduction

*Helicobacter pylori* is a widespread gastric pathogen associated with several gastrointestinal disorders, including chronic gastritis, peptic ulcers, and gastric adenocarcinoma^1^. Its remarkable ability to survive in the acidic environment of the human stomach mainly relies on urease production, biofilm formation, and other virulence factors, which collectively facilitate colonization. These mechanisms also play a key role in the development of antibiotic resistance^2,3^. The standard treatment for *H. pylori* infection consists of a combination of proton pump inhibitors (PPIs), antibiotics, and bismuth compounds. However, the efficacy of these regimens has significantly declined in recent years due to the increasing prevalence of antibiotic-resistant strains. According to the World Health Organization (WHO), resistance to key antibiotics such as clarithromycin and metronidazole has exceeded the critical threshold of 15% in many regions^4^. In particular, clarithromycin resistance has risen from approximately 15.6% in the early 2000s to nearly 40% in recent years, while metronidazole resistance has increased from 57.8 to 77.5% over the same period^5^. In addition to antimicrobial resistance, the limited stability of conventional antibiotics under gastric conditions contributes to treatment failure and recurrence of *H. pylori* infection^6^. Moreover, the increasing resistance to fluoroquinolones and other commonly used antibiotics continues to compromise eradication success rates, posing a significant challenge to current therapeutic strategies^7^. Given these challenges, natural components such as *Mentha piperita* essential oil, rich in menthol as the primary bioactive compound, have shown antimicrobial and antioxidant activities^8–10^. However, translating these promising biological effects into effective therapeutic applications remains challenging, as the unfavorable physicochemical characteristics of essential oils significantly limit their performance^9^. Previous studies have demonstrated the antibacterial, antibiofilm, and anti-inflammatory activities of ginger extract and eugenol (clove) essential oils against *H. pylori*. However, these investigations have predominantly relied on bulk essential oil^11,12^. Such natural oils are often limited by poor solubility, instability, and rapid degradation, which reduce bioavailability and lead to inconsistent antimicrobial performance^13^. To overcome these limitations, nanoemulsion-based delivery systems have emerged as a promising strategy. By reducing droplet size to the nanometer scale, nanoemulsions significantly enhance the dispersion of hydrophobic compounds in aqueous media, thereby improving solubility. They also increase the stability of essential oils by protecting them from degradation and volatilization. In addition, nanoemulsions enhance bioavailability and interaction with bacterial cells. These properties can lead to improved antimicrobial efficacy compared to bulk essential oils^14,15^. Moreover, the rational design and optimization of nanoemulsions have been shown to play a critical role in achieving desirable characteristics, including stability and controlled drug release^16^. In parallel, growing evidence from biomedical applications indicates that nanoemulsion-based delivery systems can significantly improve drug solubility, tissue penetration, and therapeutic outcomes, as demonstrated in inflammatory skin disorders such as psoriasis^17^. Recent studies have also reported a growing shift in antimicrobial therapy toward green science principles and sustainable formulations. In this context, plant-derived metabolites incorporated into nanoscale systems or bio-composites have demonstrated enhanced, or at least comparable, antibacterial and pharmacological activities against resilient microbial pathogens^18–20^. In addition to direct bactericidal effects, nanoscale systems have been shown to interfere with complex microbial processes such as quorum sensing and biofilm formation, which are critical for bacterial persistence^21^. Although several studies have highlighted the antimicrobial potential of essential oil–based nanoemulsions, most have primarily focused on planktonic bacterial cells or non-gastric pathogens. For instance, cinnamon oil nanoemulsion have been reported to exhibit superior antibacterial activity against *Klebsiella pneumoniae* compared to their bulk form^22^. Similarly, *Zanthoxylum bungeanum pericarp* essential oil nanoemulsion has also demonstrated notable antibacterial effects against foodborne pathogenic bacteria relative to its bulk counterpart^23^. In another study, turpentine essential oil nanoemulsion showed strong antibiofilm activity against methicillin-resistant *Staphylococcus aureus* (MRSA)^24^. Furthermore, even in studies targeting *H. pylori*, the emphasis has largely been placed on formulation development and physicochemical characterization, or on bulk essential oils, rather than comprehensive biological evaluation. For example, Moradialvand et al. reported enhanced antibacterial activity of *Cinnamomum zeylanicum* essential oil nanoemulsions against *H. pylori*, but did not investigate key virulence factors such as urease activity or biofilm formation and eradication^25^. Similarly, Hidalgo et al. showed that curcumin nanoformulations reduced bacterial growth and biofilm formation; however, no direct comparison with the bulk compound or evaluation of urease activity was performed^26^. In another study, Mosallam et al. developed a curcumin–clarithromycin nanoemulsion with improved antibacterial and antibiofilm effects, but without investigating urease inhibition or applying a multi-endpoint biological approach^27^. In addition, although *Mentha*-derived essential oils have shown anti-*H. pylori* activity, most studies have investigated them in bulk form rather than nanoemulsion-based delivery systems^9^. Taken together, these studies indicate that the potential of nanoformulated *Mentha piperita* essential oil to target multiple virulence pathways of *H. pylori* remains insufficiently explore. Therefore, the present study contributes to this gap by developing and physicochemically characterizing a *Mentha piperita* essential oil nanoemulsion and evaluating its antibacterial activity against *H. pylori* using a multi-target approach. Unlike previous studies, we investigate planktonic growth inhibition, urease activity suppression, and both biofilm inhibition and eradication, alongside a direct comparison with the bulk essential oil. Furthermore, the use of high-resolution imaging techniques, including scanning electron microscopy (SEM) and atomic force microscopy (AFM), provides deeper insight into the structural and mechanistic effects of the nanoemulsion on *H. pylori* cells (Table 1).

**Table 1:** Different formulation of Mentha piperita nanoemulsion.

Nanoemulsion with 4% MPEO	MPEO: Tween80 (v/v)	MPEO: Tween 80 (µL)	Water (µL)	Sonication time (Minutes)	Amplitude (%)	Pulse cycle(s)		Total energy input (J)
						ON	OFF	
MPNE-100	1:1	400: 400	9200	7	80	30	30	17,640
MPNE-200	1:2	400: 800	8800	7	80	30	30	17,640
MPNE-300	1:3	400: 1200	8400	7	40	30	30	8841
MPNE-400	1:4	400: 1600	8000	7	40	30	30	8841

MPEO, *Mentha piperita* essential oil; MPNE, *Mentha piperita* nanoemulsion.The total volume of each formulation was adjusted to 10 mL.

## Result

### Characterization of optimized nanoemulsion

**Fig. 1:**
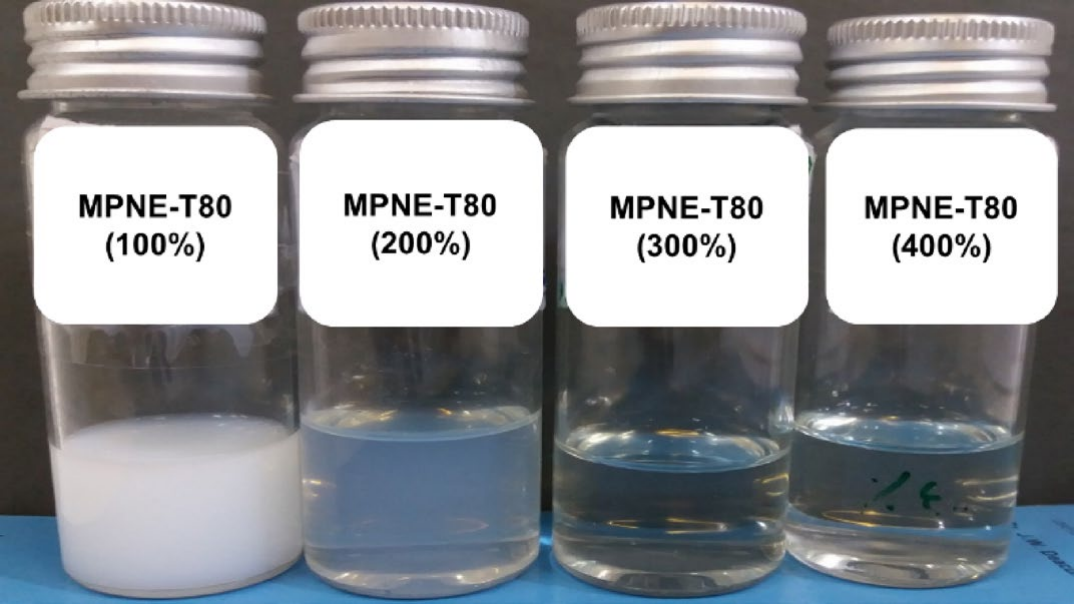
Visual appearance of *Mentha piperita* nanoemulsions prepared with different Tween 80 ratios (essential oil 4%).

The physical appearance of nanoemulsions prepared with varying Tween 80 ratios is shown in Fig. 1. The MPNE-100 appeared whitish and non-transparent with evident phase separation, whereas the MPNE-200 formulation exhibited a pale bluish tint and slight turbidity. In contrast, the MPNE-300 and MPNE-400 formulations were visually transparent and physically stable, indicating the formation of a well-dispersed nanoemulsion system. Based on optimal visual clarity and reduced surfactant content, MPNE-300 was selected for further optimization using co-surfactants. Formulations containing different co-surfactants were further evaluated based on macroscopic appearance and stability. The SDS optimization revealed that 0.025 (w/v) provided optimal stability with minimal foaming (Fig. S1 in Supplementary Information). For the Tween 80: PEG 400 ratio, 2:1 resulted in a slightly bluish appearance, whereas 1:2 produced a turbid and unstable system (Fig. S2 in Supplementary Information). Accordingly, the 2:1 ratio was selected for subsequent characterization. Physicochemical characterization revealed that the MPNE stabilized with PEG showed rapid instability after sonication. Although the initial droplet size (Z-average: 210 ± 34 nm) and PDI (0.29 ± 0.01) could be measured immediately, macroscopic aggregation and turbidity developed within 1 h, preventing reliable subsequent measurements. By comparison, as shown in Table 2, the freshly prepared MPNE stabilized with SDS (MPNE–SDS) exhibited a relatively monodisperse nanosystem with a zeta potential (–30.9 ± 1.3 mV), indicating strong electrostatic repulsion between droplets. During storage at 25 °C, no significant changes in droplet size or PDI were observed after 3 months, indicating good stability. After 6 months, a significant increase in droplet size was detected, suggesting partial aggregation; however, no visible phase separation, creaming, or sedimentation occurred, and the nanoemulsion remained transparent. Given its transparency, monodispersity, and overall stability, MPNE–SDS was selected for subsequent biological assays.

**Table 2:** Physicochemical stability of MPNE-SDS formulations during storage at 25 °C.

Parameter	MPNE-SDS (Fresh)	MPNE-SDS (3 month)	MPNE-SDS (6 month)
Z-average diameter (nm)	168.34 ± 8.20^a^	182.43 ± 7.17^a^	414.33 ± 13.29^b^
PDI	0.28 ± 0.03^a^	0.33 ± 0.02^a^	0.35 ± 0.01^a^
Visual appearance	Transparent	Transparent	Transparent
Phase separation	None	None	None

MPNE-SDS, *Mentha piperita* nanoemulsion stabilized with sodium dodecyl sulfate; Values are mean ± SD (*n* = 3);Groups with different letters are significantly different (*p* < 0.001, Repeated measures one-way ANOVA, Tukey’s post-hoc test).

### Antioxidant effect

MPNE-SDS showed higher radical-scavenging activity (56.33 ± 0.71%) and Trolox equivalent antioxidant capacity (554.7 ± 8.31 µmol Trolox) compared to MPEO (24.03 ± 1.40% and 179 ± 16.21 µmol Trolox, respectively; *p* < 0.05, Student’s t-test). In contrast, the blank nanoemulsion exhibited negligible radical-scavenging activity (< 3%). Notably, the approximately threefold increase in Trolox equivalent antioxidant capacity observed for MPNE-SDS compared to MPEO suggests that the improvement is not only statistically significant but also biologically meaningful, reflecting enhanced antioxidant performance following nanoemulsification.

### Toxicity to cells

**Fig. 2:**
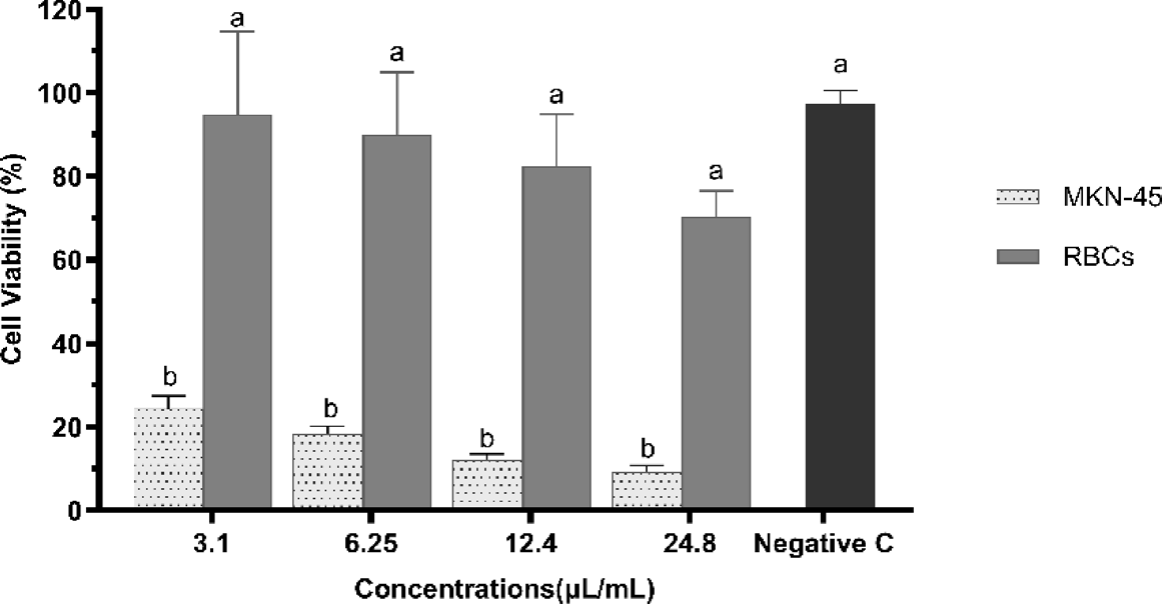
Cytotoxicity of MPNE–SDS on MKN-45 cancer cells and sheep red blood cells (RBCs, primary cells) after 24 h. Data are expressed as % cell viability ± SD (*n* = 3). Different letters indicate statistically significant differences (Dunnett’s test, *p* < 0.001).

The cytotoxicity of MPNE–SDS was assessed on both MKN-45 cancer cells and primary RBCs by measuring cell viability after 24 h of exposure (Fig. 2). The nanoemulsion exhibited low toxicity toward RBCs, with cell viability remaining above 70% at all tested concentrations, indicating its relative biocompatibility. In contrast, MPNE–SDS reduced the viability of MKN-45 cells in a concentration-dependent manner, showing a statistically significant difference compared to the control. Cell viability decreased from 24.4 ± 3.1% at the lowest concentration to 9.2 ± 1.5% at the highest concentration. In addition, the IC₅₀ value for MKN-45 cells was determined to be below 3.12 µL/mL. Tween 80 and SDS alone did not exhibit cytotoxic effects.

### Antimicrobial activity

**Fig. 3:**
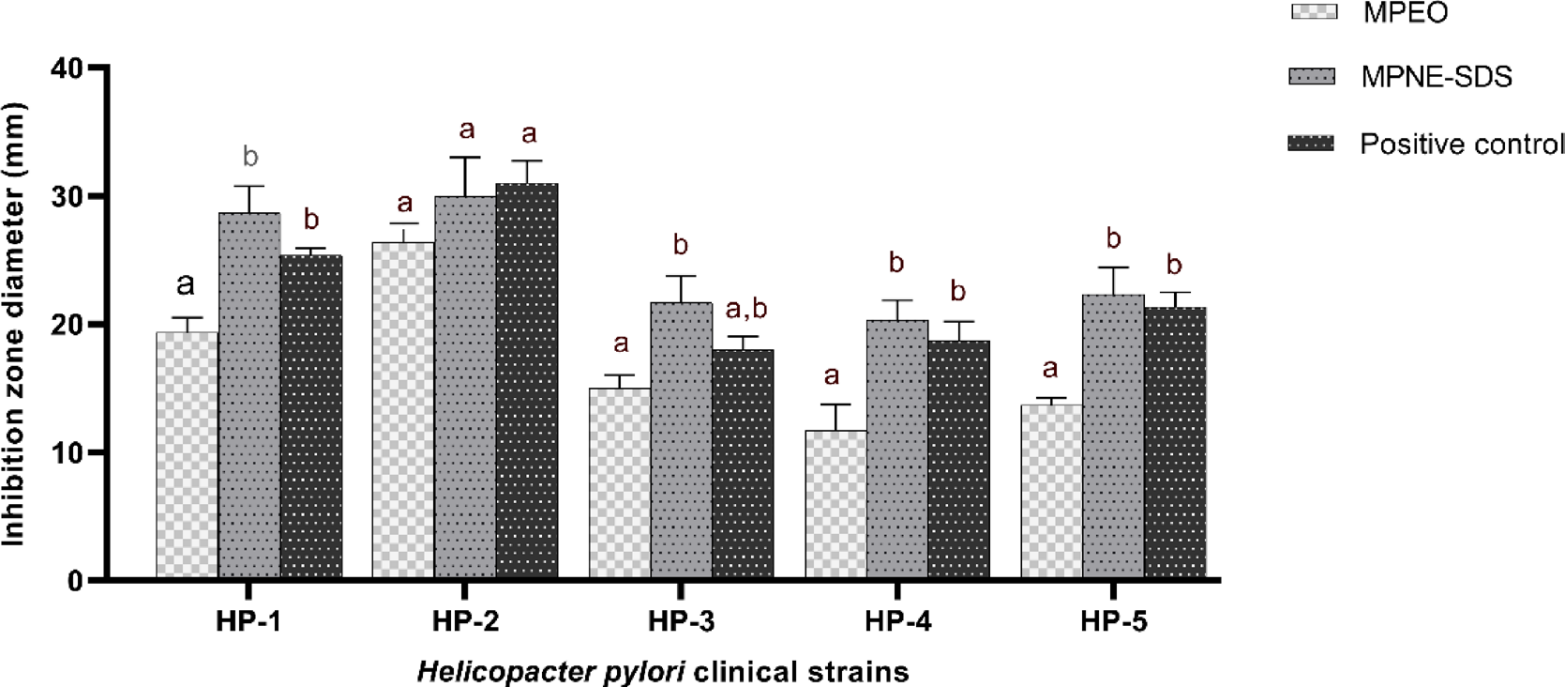
Antibacterial activity of MPNE-SDS and MPEO against *H. pylori* strains. Positive control (Amoxicillin), and negative control (Tween 80 and SDS). Data represent the mean ± SD. Different letters indicate statistically significant differences between treatments (*p* < 0.05, one-way ANOVA with Tukey’s post hoc).

The antimicrobial activity of MPNE-SDS and its essential oil against *H. pylori* strains is shown in Fig. 3. The GC-MS analysis of MPEO and the antibiotic resistance profiles are reported in Supplementary Tables S1 and S2, respectively. The MPNE–SDS formulation produced significantly larger inhibition zones than MPEO, except for the HP2 strain, where no significant difference was observed. This result confirms the improved antibacterial efficacy of MPEO after nanoemulsification. The inhibition zone diameters of MPNE–SDS ranged from 20.3 ± 1.5 to 30.6 ± 3.2 mm, whereas those of MPEO ranged from 11.6 ± 2.0 to 26.3 ± 1.5 mm, indicating strain-dependent differences in susceptibility. The positive control (amoxicillin) showed a variable inhibitory effect, with inhibition zones ranging from 18 ± 1 to 31 ± 1.7 mm. In contrast, the negative control (Tween 80 and SDS) showed no inhibition (0 mm). Notably, no statistically significant difference was observed between MPNE–SDS and the positive control (amoxicillin) across all tested strains, indicating comparable inhibition zone diameters under our experimental conditions. Statistical analysis further significant effects of both treatment and strain, as well as their interaction. Consistent with IZ results, MIC determination further demonstrated that formulation of MPEO into a nanoemulsion markedly enhanced its antibacterial potency, achieving bacterial growth inhibition at lower concentrations (6.25–50 µL/mL) compared with the essential oil (up to 100 µL/mL) (Table 3).

**Table 3:** Minimum inhibitory concentration (MIC) values of MPNE-SDS and its essential oil against clinical H. pylori isolates.

H. pylori isolate	MPEO (approx. µg/mL)	MPEO (µL/mL)	MPNE-SDS (µL/mL)
HP-1	11,225 ± 0	12.5 ± 0	6.25 ± 0
HP-2	22,450 ± 0	25 ± 0	12.5 ± 0
HP-3	> 89,800	> 100	50 ± 0
HP-4	89,800 ± 0	100 ± 0	25 ± 0
HP-5	44,900 ± 0	50 ± 0	25 ± 0

MPNE-SDS, *Mentha piperita* nanoemulsion stabilized with sodium dodecyl sulfate. MPEOl, *Mentha piperita* essential oil. All values are mean ± standard deviation of three replicates.

### Vapor diffusion tests

In the vapor-phase test, MPEO demonstrated the highest antibacterial activity, generating inhibition zones that fully covered the plates (Table 4). In comparison, sonicated MPNE–SDS displayed moderate inhibitory effects, while the non-sonicated formulation showed no detectable activity. This result indicates that sonication improves the antimicrobial performance of the nanoemulsion.

**Table 4:** The inhibition zone diameter (mm) of MPEO and MPNE-SDS in vapor phase against H. pylori isolates.

Strains	MPEO	MPNE-SDS (Sonicated)	MPNE-SDS (Non-sonicated)	Negative control (Tween80 and SDS)
HP-3	> 80.0^a^	61.3 ± 1.1^b^	–	–
HP-4	> 80.0^a^	49 ± 3.6^b^	–	–
HP-5	> 80.0^a^	70.6 ± 1.1^b^	–	–

(–): no effect, MPNE-SDS *Mentha piperita* nanoemulsion stabilized with sodium dodecyl sulfate, MPEO *Mentha piperita* essential oil Values are mean ± SD (*n* = 3) Different letters indicate statistically significant differences (paired t-test, *p* < 0.05)

### Atomic force microscopy (AFM)

**Fig. 4:**
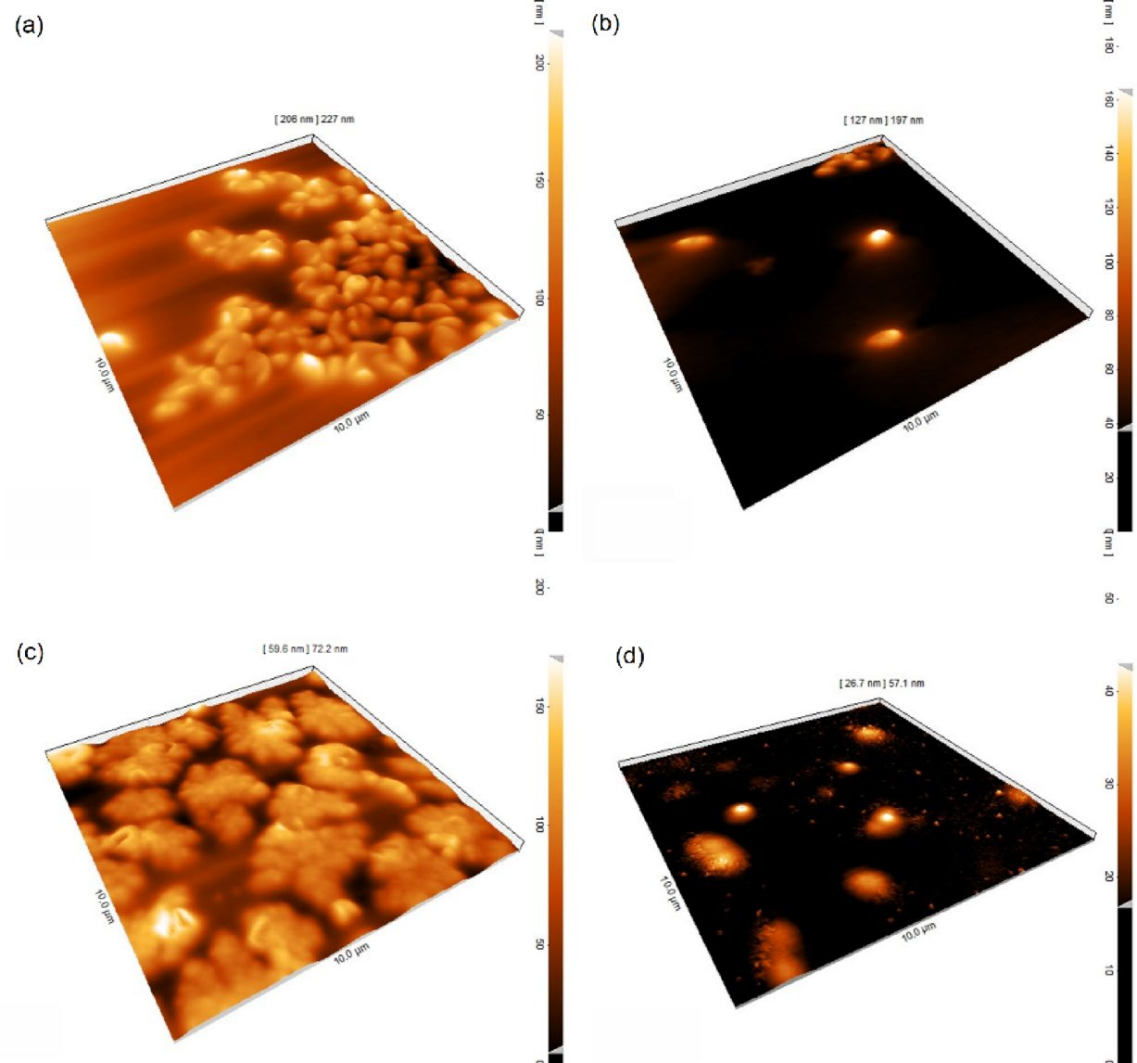
Atomic force microscopy (AFM) images illustrating the morphological effects of MPNE–SDS on *H. pylori*. (**a**, **b**) untreated control; (**c**, **d**) treated with sample.

Using AFM, the antibacterial activity of MPNE-SDS against *H. pylori* was visualized through high-resolution three-dimensional images (Fig. 4). AFM clearly revealed distinct morphological differences between untreated and MPNE-SDS–treated cells. In the control samples (Fig. 4a,b), bacterial cells exhibited the characteristic helical rod-shaped morphology with smooth, continuous surfaces. AFM height analysis showed peaks at 227 and 197 nm, indicating well-preserved cellular integrity. In contrast, MPNE–SDS–treated cells (Fig. 4c,d) exhibited clear evidence of membrane breach and shrinkage. The cell surfaces appeared irregular and disrupted, with pronounced flattening and collapse on the substrate, showing peaks at 72.2 and 57.1 nm, which confirm severe structural damage and cell lysis. In addition, depressions observed on the cell surfaces demonstrate the shape alterations induced by MPNE-SDS. AFM findings are consistent with the antibacterial assay results, confirming that MPNE-SDS exerts its bactericidal activity.

### Whole-cell urease activity

MPNE–SDS significantly reduced whole-cell urease activity compared with the untreated control (one-way ANOVA followed by Tukey’s post-hoc test; *p* < 0.05), increasing from 16.4 ± 5.3% at ½ MIC to 44.4 ± 10.6% at MIC, and then reaching a plateau at 57.7 ± 4.7% and 65.2 ± 1.7% at 2× and 4× MIC, respectively. No significant difference was observed between the two highest concentrations (*p* > 0.05). The IC₅₀ was calculated as 41.6 µL/mL (95% IC: 32.2–55.4 µL/mL).

### Effect of pH on the anti-*H. pylori* activity of MPNE-SDS.

**Fig. 5:**
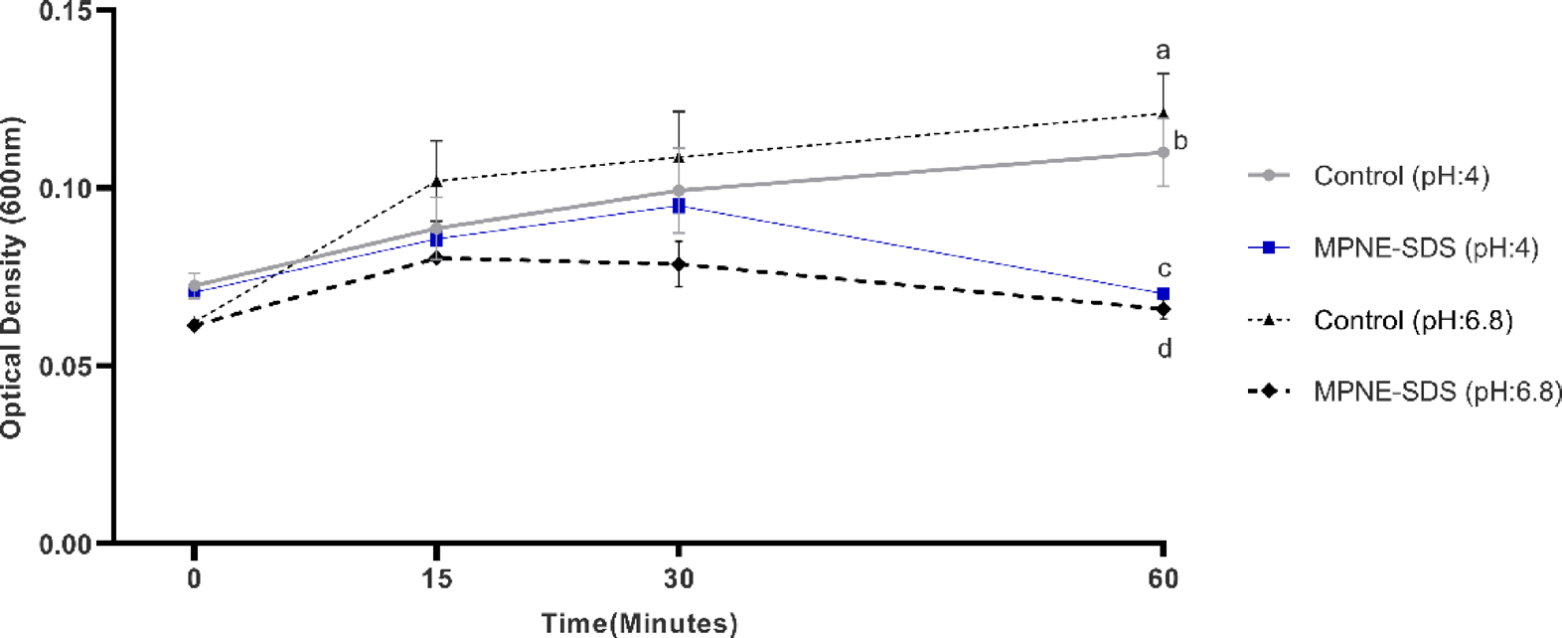
Effect of MPNE-SDS on *H. pylori* growth under acidic (pH 4) and neutral (pH 6.8) conditions. Growth was measured by OD600 at 0, 15, 30, and 60 min. Data represent mean ± SD (*n* = 3). Statistical differences at 60 min between groups are indicated by different letters (*p* < 0.05, Two-way ANOVA followed by Tukey’s test).

MPNE-SDS significantly inhibited the growth of *H. pylori* after 60 min at both pH 4 and 6.8, with a markedly stronger inhibitory effect observed under acidic conditions (pH 4) (Fig. 5). No significant differences were observed at 0, 15, or 30 min at either pH. Control groups showed no significant differences between pH conditions at any time point.

### Anti-biofilm activity

**Fig. 6:**
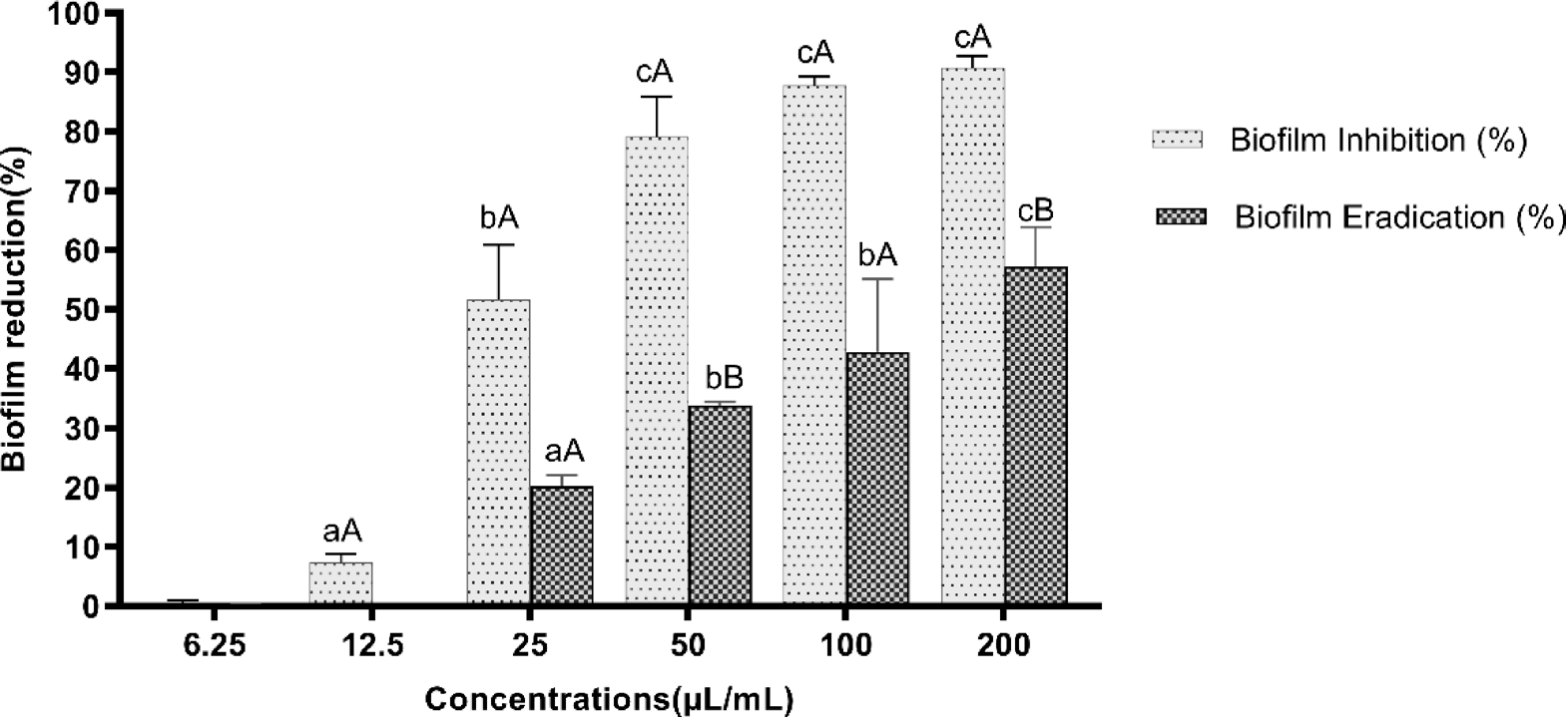
Biofilm inhibition and eradication of *H. pylori* by MPNE-SDS at different concentrations. Data are mean ± SD (*n* = 3). Different lowercase letters indicate differences among concentrations; different uppercase letters indicate differences between inhibition and eradication at the same concentration (Šídák’s test, *p* < 0.05).

**Fig. 7:**
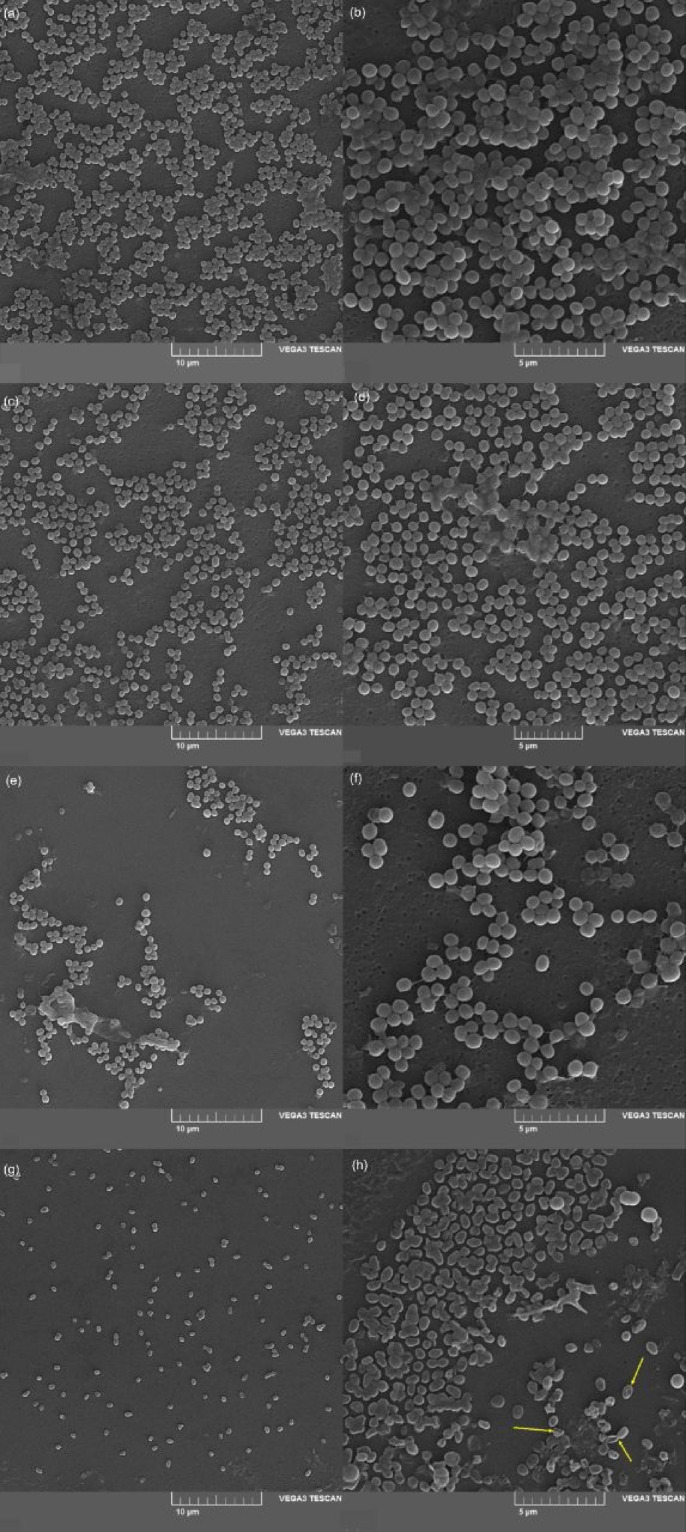
Scanning electron microscopy images showing *H. pylori* biofilm inhibition by MPNE–SDS: Untreated control (**a**, **b**) ½ MIC (**c**, **d**) MIC (**e**, **f**) and 4× MIC (**g**, **h**). Yellow arrows indicate cavities and damaged bacterial cells.

As shown in Fig. 6, nanoemulsion inhibited *H. pylori* biofilm with increasing concentrations, with significant reduction observed from 25 µL /mL onwards and over 90% inhibition at 200 µL /mL (51.6 ± 9.2–90.6 ± 2.08%). In contrast, biofilm eradication required higher concentrations to achieve noticeable effects. At 25 µL/mL, biofilm eradication remained below 25%, whereas treatment with 200 µL/mL increased eradication to approximately 60%, corresponding to values ranging from 20.2 ± 1.7 to 57.2 ± 6.6%. The IC₅₀ for biofilm inhibition was 24.48 µL/mL, while the EC₅₀ for biofilm eradication was 133.1 µL/mL, indicating that MPNE-SDS is more effective at preventing biofilm formation than at eliminating established biofilms. SEM imaging (Fig. 7) further confirmed the inhibitory effects of MPNE–SDS on *H. pylori* biofilm formation, in agreement with quantitative biofilm inhibition data. The untreated control (Fig. 7a,b) exhibited a dense, compact biofilm with tightly aggregated coccoid cells embedded in a well-developed extracellular polymeric matrix (EPS), indicating biofilm maturity and structural stability. At ½ MIC (25 µL/mL), corresponding to 51.60% biofilm inhibition, the biofilm showed initial structural disruption, characterized by matrix loosening and increased intercellular spacing (Fig. 7c,d). At MIC (50 µL/mL), where biofilm inhibition reached 78.73%, a marked reduction in biofilm density and degradation of the EPS layer were observed, with only scattered bacterial clusters remaining (Fig. 7e,f). At 4×MIC (200 µL/mL), associated with 90.67% inhibition, the biofilm structure was almost completely collapsed, and many cells exhibited ruptured morphology (Fig. 7g,h). These findings demonstrate a clear correlation between increasing MPNE–SDS concentration, higher percentages of biofilm inhibition, and progressive structural damage, suggesting its dual antibiofilm and bactericidal activity.

## Discussion

In this study, we successfully prepared a *Mentha piperita* essential oil–based nanoemulsion stabilized with SDS (MPNE-SDS) and evaluated its physicochemical stability and antibacterial effects against *H. pylori* under in vitro conditions. The nanoemulsion displayed a uniform droplet size below 200 nm and a favorable zeta potential, ensuring efficient dispersion and maintaining stability over three months. These features are consistent with previous reports showing that nanoemulsions enhance physical stability by forming interfacial films that minimize droplet coalescence and aggregation^28–30^. Similarly, studies on Pickering emulsions have demonstrated improved stability and physicochemical properties of bioactive-loaded emulsions through effective interfacial stabilization^31^. Compared with the bulk essential oil, MPNE-SDS exhibited greater antibacterial efficacy, as indicated by lower MIC values and larger inhibition zones against most *H. pylori* isolates. Since MPEO is poorly soluble in water, higher doses are generally required for bacterial inhibition. Nanoemulsification likely enhances the solubility, diffusion, and bioavailability of MPEO’s hydrophobic constituents, enabling antibacterial effects at lower concentrations. These findings align with previous research showing that *Mentha piperita* (peppermint) nanoemulsions outperform the bulk essential oil in antibacterial performance^32,33^. The improved efficacy is likely due to the small droplet size, which increases surface area and promotes diffusion across bacterial membranes. Additionally, emulsifier droplets may interact with bacterial membranes, facilitating localized release of essential oil components. Electrostatic interactions between positively charged nanoemulsion droplets and negatively charged bacterial cell walls may further increase the local concentration of active compounds, enhancing antimicrobial effects^27,34,35^.

Interestingly, in vapor-phase assays, the bulk MPEO showed greater inhibitory effects, supporting the well-established antimicrobial potential of volatile monoterpenes^36,37^. These results indicate that while nanoemulsions are advantageous for contact-dependent antibacterial activity, unformulated essential oils may be more effective in vapor-phase conditions, likely due to their higher volatility^38^. In addition to its antibacterial effects, the nanoemulsion maintained activity against *H. pylori* under both acidic (pH 4) and neutral (pH 6.8) conditions, suggesting that the formulation remains stable and functionally active under simulated gastric environments. However, these in vitro findings should be interpreted with caution, as the complex biological conditions of the human stomach—including gastric enzymes, dynamic pH variations, and the mucus barrier—may significantly influence nanoemulsion behavior and efficacy in vivo. These observations are consistent with previous reports showing that nanoemulsion systems can protect encapsulated bioactive compounds from acid-induced degradation, thereby enhancing local antibacterial effectiveness^27,39^. Moreover, the nanocarrier system of MPNE–SDS enhances antioxidant capacity likely by protecting phenolic compounds from oxidative degradation and improving their dispersibility in aqueous media, which increases radical-scavenging activity^26,40^. Supporting this observation, Zamaniahari et al. reported that *Mentha spicata* nanoemulsions showed higher antioxidant activity than the bulk essential oil using the DPPH assays^41^. The approximately threefold increase in Trolox equivalent antioxidant capacity observed for MPNE-SDS (554.7 ± 8.31 µmol Trolox) relative to MPEO (179 ± 16.21 µmol Trolox) indicates a substantial enhancement following nanoemulsification. Similar trends have been reported in other nanoformulation studies, including Neem oil nanoemulsions (~ 23–53 mg Trolox) versus free Neem oil (7.6 mg Trolox), as well as curcumin-loaded nanoformulations (> 50,000 µmol Trolox/µmol curcumin) compared to free curcumin (46 µmol Trolox/µmol curcumin)^26,42^. Overall, these findings demonstrate that nanoencapsulation improves antioxidant performance. This combined antibacterial–antioxidant functionality further suggests the multifunctional potential of the formulation.

AFM imaging (Fig. 4) provided further mechanistic insights into the antibacterial activity of MPNE–SDS. The observed membrane rupture and surface roughening suggest a membrane-disruptive mode of action^43^. These structural alterations likely increase membrane permeability, thereby facilitating the intracellular delivery of peppermint-derived phenolics to enzymatic targets such as urease. As urease catalyzes the hydrolysis of urea into ammonia, a critical step for *H. pylori* survival in the acidic gastric environment, its reduction represents an important antibacterial-related effect^44^. The formulation exhibited a marked reduction in whole-cell urease activity, with an IC₅₀ of 41.6 µL/mL. This effect is comparable to that reported for other nanoformulations, including silver-based and fucoidan-coated systems^45,46^. Furthermore, previous in silico studies have suggested that urease inhibition by small molecules may involve interactions within the active site^47^; however, in the present whole-cell system, the observed reduction is more likely attributable to a combination of antibacterial activity and potential functional modulation of urease activity, rather than confirmed direct enzyme binding. Viability assessment also confirmed that *H. pylori* cells remained detectable under all experimental conditions, although reduced growth was observed at higher concentrations of MPNE–SDS. Notably, residual bacterial viability may still be present even at 4×MIC, indicating that the observed reduction in urease activity cannot be attributed solely to complete bacterial eradication and may also reflect functional modulation of urease activity under whole-cell conditions.

Importantly, MPNE–SDS effectively inhibited *H. pylori* biofilm formation, a major factor contributing to antibiotic resistance and chronic colonization^48^. Notably, significant inhibition was observed even at sub-MIC levels, in agreement with previous reports describing concentration-dependent antibiofilm effects of nanoemulsions^26^. This effect likely arises from interference with key stages of biofilm development. The nanoscale droplets increase the contact surface of bioactive compounds with bacterial cells, thereby reducing initial adhesion and aggregation mediated by adhesins such as *BabA*, and disrupting early biofilm formation. In addition, enhanced delivery of peppermint phenolics may interfere with quorum sensing and other regulatory pathways involved in biofilm maturation, leading to reduced synthesis of extracellular polymeric substances (EPS) and virulence factors essential for structural integrity^45,49^. SEM analysis revealed that coccoid forms, associated with increased antibiotic tolerance and biofilm-associated dormant states, were observed in the control samples (Fig. 7a,b)^50,51^. At higher MPNE–SDS concentrations, biofilm formation was markedly reduced, accompanied by collapse of the EPS matrix and the appearance of flattened or ruptured bacterial cells. These morphological changes indicate that the nanoemulsion not only inhibits biofilm formation but also exerts direct bactericidal effects. The observed membrane damage is likely due to surfactant-induced destabilization, leading to increased permeability and eventual cell lysis^25,52–54^. These findings align with previous studies on essential oil-loaded nanoemulsions^55–57^. Moreover, recent evidence highlights that sustainable nanoformulations can disrupt bacterial communication pathways, such as quorum sensing, which are critical for biofilm integrity^31^. Supporting this, other nanoformulations have demonstrated comparable antibiofilm activity against *H. pylori*. For instance, a nanoemulsion based on *Casearia sylvestris* extract reduced bacterial viability and biofilm formation both in vitro and in vivo, partly through terpenoid-induced membrane damage and anti-inflammatory effects^58^. Likewise, a curcumin–azithromycin nanoemulsion inhibited biofilm formation, downregulated virulence genes (*babA*,* hopQ*,* ureA*), and enhanced antibiotic efficacy via improved local delivery^59^. Collectively, these findings suggest the potential of nano-based strategies in preventing *H. pylori* biofilm formation.

Beyond its antimicrobial and antibiofilm properties, it was important to assess whether MPNE–SDS affects the viability of human gastric cancer cells (MKN-45) and normal primary cells (RBCs) at the tested concentrations. The results showed an IC₅₀ below 3.12 µL/mL for MKN-45 cells, indicating a pronounced cytotoxic effect at concentrations lower than those required for antibacterial and antibiofilm activity. Although this suggests a partial overlap between cytotoxic and antimicrobial ranges, the formulation demonstrated a degree of selectivity. Specifically, MPNE–SDS reduced the viability of MKN-45 cells, while RBCs remained largely unaffected, maintaining viability above 70% even at the highest tested concentrations, indicating relative biocompatibility with normal cells. This differential response may be attributed to fundamental differences in cellular uptake and metabolic activity. Cancer cells typically exhibit elevated metabolic rates, altered membrane composition, and increased susceptibility to oxidative stress^60^, which may enhance nanoemulsion uptake and intracellular release. In contrast, RBCs, lacking nuclei and mitochondria^61^, are less susceptible to apoptosis-mediated cytotoxicity, contributing to their preserved viability following treatment. Moreover, the higher concentrations required for antibacterial activity, compared to the IC₅₀ for cancer cells, likely reflect the structural complexity of bacterial cell walls and biofilm matrices. These act as physical and diffusional barriers that limit antimicrobial penetration and necessitate higher effective doses^62^. In this context, plant-derived bioactive formulations have been reported to retain biological activity while exhibiting acceptable biocompatibility and hemocompatibility, supporting the compatibility profile observed for the present nanoemulsion system^63^. Recent studies on plant-derived nanoemulsion and nanoparticle systems have also demonstrated favorable biocompatibility and environmental safety profiles, further supporting the safe application of such formulations^64,65^. Importantly, Tween 80 and SDS alone showed no cytotoxic effects, indicating that the observed activity in cancer cells is primarily attributable to the bioactive components of MPEO, such as menthol and menthone, which have been reported to induce pro-apoptotic effects in various cancer cell lines^66–68^. Overall, these findings highlight the importance of careful dose optimization to define a precise therapeutic window and emphasize the need for further in vivo studies to evaluate the systemic safety, pharmacokinetics, and therapeutic efficacy of MPNE–SDS prior to clinical application.

### Future perspectives

Although the present study demonstrated the antibacterial, anti-virulence, and preliminary safety profile of the *Mentha piperita* essential oil nanoemulsion, further biosafety evaluation is required to strengthen its translational potential. Future investigations should include expanded cytocompatibility assessments across additional mammalian cell lines, along with hemolysis and erythrocyte membrane integrity assays, as well as analyses of oxidative stress and apoptosis-related pathways. In addition, comprehensive in vivo safety evaluation in appropriate animal models will be essential to confirm its therapeutic potential.

## Conclusion

This study suggests the potential of MPNE–SDS as a multifunctional agent against *H. pylori* under in vitro conditions. The optimized formulation exhibited physicochemical stability, potent antibacterial and antibiofilm activities, and retained its effectiveness under acidic gastric-like conditions. These observations indicate that the nanoemulsion system can disrupt bacterial integrity and interfere with key survival mechanisms, including urease activity. MPNE–SDS also showed selective cytotoxicity toward gastric cancer cells while maintaining relative biocompatibility with normal primary cells, suggesting a favorable preliminary safety profile. Despite these encouraging results, several limitations should be acknowledged. The current findings are restricted to in vitro conditions and may not fully capture the complexity of the in vivo gastric environment. In particular, the long-term stability of the nanoemulsion in the presence of gastric enzymes, as well as its controlled release behavior within the gastric mucus layer, remain to be elucidated. Moreover, critical parameters such as bioavailability, pharmacokinetics, long-term safety, and host interactions require systematic investigation. Taken together, these findings support further preclinical investigation of *Mentha piperita*-based nanoemulsion as a potential adjunct to antibiotic therapy for effective *H. pylori* eradication. Future research should prioritize comprehensive in vivo validation, along with advanced biocompatibility assessments, to fully establish the translational potential and therapeutic value of this formulation.

## Materials and methods

### Materials

*Mentha piperita* essential oil (MPEO) was from Tabib Daru Co. (Tehran, Iran). Tween 80 (Ibersco Co., Tehran, Iran), sodium dodecyl sulfate (SDS), and polyethylene glycol 400 (PEG 400) (Merck-Millipore, Germany) were used as surfactants and cosurfactants. Brucella agar was obtained from Merck (Germany), and defibrinated sheep blood was supplied by Darvash Co. (Tehran, Iran). The human gastric cancer cell line MKN-45 was obtained as a live culture from the Biological and Genetic Resources Centre of Iran. RPMI 1640 medium (BioIdea Co., Iran), fetal bovine serum (FBS) for cell culture (Zisera Co., Iran), and the MTT assay kit (3-(4,5-dimethylthiazol-2-yl)-2,5-diphenyltetrazolium bromide; Cib Biotech Co., Tehran, Iran) were used for cell-based experiments. DPPH (2,2-diphenyl-1-picrylhydrazyl; Kavosh Arian Azma Co., Birjand, Iran) was used as the antioxidant assay reagent.

### Methods

#### Preparation and characterization of optimized nanoemulsion

Nanoemulsions containing 4% (v/v) MPEO were prepared using a high-energy ultrasonic technique^69^. The surfactant-to-essential-oil ratio was varied as 1:1, 2:1, 3:1, and 4:1 (v/v), which were labeled as 100, 200, 300, and 400 *Mentha piperita* nanoemulsion (MPNE), respectively. Initially, Tween 80 and distilled water were pre-mixed using a magnetic stirrer (Pole Ideale Pars Co., Iran) at 600 rpm for 15 min. The essential oil was then added dropwise (≈ 1 drop min⁻¹) while stirring was continued for an additional 30 min to ensure homogeneity. The pre-emulsion was subsequently sonicated using an ultrasonic processor (MISONIX S-4000, USA) with the 10 mm probe positioned approximately 2 cm from the vessel bottom. During sonication, samples were maintained in an ice bath to avoid excessive heating. Formulation details are summarized in Table 1. The MPNE-300 (containing 400 µL of MPEO, 1200 µL of Tween 80, and 8400 µL of water in a total volume of 10 mL nanoemulsion) was further optimized using two co-surfactants, SDS and PEG 400, as described above to enhance stability^57,70,71^. The optimization of the co-surfactant system was conducted using a one-factor-at-a-time (OFAT) approach. The SDS concentration was optimized by screening four levels: 0.012, 0.025, 0.05, and 0.1 (w/v). The surfactant-to-co-surfactant ratio (Tween 80: PEG 400) was also screened at two volume ratios: 1:2 and 2:1 (v/v). The final optimized formulation, hereafter referred to as MPNE-SDS, was characterized for droplet size and polydispersity index (PDI) by dynamic light scattering (DLS, VASCO2, Cordouan Tech, France). The nanoemulsions were diluted 1:10 (v/v) with deionized water prior to measurement to minimize multiple scattering effects. Measurements were conducted at 25 °C, with refractive index and viscosity parameters set at 1.331 and 0.888 cP, respectively. Surface charge was determined by zeta potential analysis (WALLIS, Cordouan Tech, France). For zeta potential measurements, samples were dispersed in deionized water (pH 6, conductivity 0.8 µS/cm) and allowed to equilibrate in the measurement cell for 120 s. Physical stability of the optimized MPNE-SDS nanoemulsion was monitored for 6 months by assessing particle size, PDI, and visual appearance (transparency) at 0, 3, and 6 months under storage at 25 °C in dark conditions^70,72^. All measurements were presented as mean ± standard deviation (SD) from three independent formulation batches.

### DPPH radical scavenging method for antioxidant activity

The radical-scavenging activity (*n* = 3) of MPNE-SDS and MPEO was assessed using the DPPH method^26^. A mixture of 10 µL sample and 250 µL DPPH solution (25 mM) was incubated in the dark for 15 min, and absorbance was recorded at 517 nm using a microplate reader (Epoch, BioTek Instruments, Inc., Winooski, VT, USA). The percentage of radical scavenging activity (RSA%) was calculated by directly comparing the absorbance of each sample with that of the blank solution containing DPPH in ethanol. A blank nanoemulsion containing SDS and Tween 80 was used as the vehicle control. A Trolox standard curve (31.25–1000 µM) was then used to express the antioxidant capacity of each formulation as Trolox equivalent (µmol Trolox per µmol of sample).

### Cellular toxicity assessment by MTT assay

The cytotoxicity of MPNE-SDS was evaluated using the [3-(4,5-dimethylthiazol-2-yl)-2,5-diphenyl tetrazolium bromide] (MTT) assay on both the MKN-45 cancer cell line and primary sheep red blood cells (RBCs)^73,74^. MKN-45 cells (passages 5–15) were cultured in RPMI-1640 medium supplemented with 20% FBS for 24 h and incubated for 24 h before treatment. Cells were seeded into 96-well plates at a density of 1 × 10⁴ cells per well and exposed to increasing concentrations of MPNE-SDS (3.1–24.8 µL/mL) for 24 h at 37 °C with 5% CO₂.

For RBCs, 10 mL of fresh defibrinated sheep blood was centrifuged at 1500 × g for 15 min. The supernatant was discarded, and the RBCs pellet was washed three times with phosphate-buffered saline (PBS). The washed RBCs were resuspended and diluted in PBS to obtain a suspension corresponding to approximately 2% hematocrit (~ 1:50 dilution of packed RBCs). 100 µL of this RBC suspension were added per well in a 96-well plate. The cells were then incubated under standard conditions (37 °C, 5% CO₂) for 24 h, followed by treatment with the same concentration range of MPNE-SDS for an additional 24 h. Following treatment, 20 µL of MTT solution (5 mg/mL) was added to each well, and the plates were incubated for 4 h. The supernatant was subsequently removed, and the resulting formazan crystals were solubilized using 100 µL of dimethyl sulfoxide (DMSO). Absorbance was measured at 570 nm using a microplate reader (Epoch, BioTek Instruments, Inc., Winooski, VT, USA). Cell viability was calculated as a percentage relative to untreated controls, while Tween 80 and SDS at equivalent concentrations were included as formulation controls. All experiments were conducted in triplicate.

### *Helicobacter pylori* strains

Five *H. pylori* clinical isolates obtained from the HPGC collection (Pasteur Institute of Iran, Tehran) were used in this study. The isolates were cultured on Brucella agar supplemented with 10% (v/v) defibrinated sheep blood, vancomycin (5 µg/mL), and amphotericin B (8 mg/L), and incubated at 37 °C under microaerophilic conditions for 3–5 days. Strains were characterized by colony morphology, Gram staining, biochemical tests, and PCR analysis targeting the specific *glmM* gene^75^. Antibiotic susceptibility was confirmed using disk diffusion method, and inhibition zones were interpreted according to the Clinical and Laboratory Standards Institute (CLSI)^76^. Subsequently, isolates were selected based on their resistance profiles. Clinical sample collection and bacterial isolation were conducted in accordance with protocols approved by the Committee on Ethical Issues in Medical Research, Pasteur Institute of Iran (Ref. No. IR.PII.REC.1394.57). Written informed consent was obtained from all patients before sample collection.

### Agar well diffusion test

The antibacterial activity of MPNE-SDS and MPEO was evaluated using the agar well diffusion method in triplicate. The turbidity of the bacterial suspension was measured using a spectrophotometer at 600 nm. The inoculum was then adjusted to approximately 3 McFarland standard, corresponding to an estimated 10⁷ CFU/mL^77^. A total of 100 µL of the standardized suspension was spread onto Mueller–Hinton agar supplemented, and 50 µL of each sample was added into wells. Tween 80 and SDS served as negative controls, while a standard antibiotic (Amoxicillin, 25 µg) was used as a positive control. Plates were incubated at 37 °C for 72 h under microaerophilic conditions, and the mean inhibition zone diameter (IZ) was measured^78^.

### Minimum inhibitory concentration (MIC)

MICs of MPNE-SDS and MPEO were determined using the agar dilution method in triplicate, according to CLSI guidelines^79^. Two-fold serial dilutions of each formulation (final concentrations ranging from 6.25 to 100 µL/mL) were incorporated into Mueller–Hinton agar supplemented with 10% (v/v) sheep blood. The bacterial inoculum was prepared as described above. Subsequently, 5 µL of the standardized suspension was applied onto the agar surface, yielding an estimated inoculum of 5 × 10^3^–5 × 10^4^ CFU per spot (~ 10⁴–10⁵ CFU/cm²). Plates were incubated under standard conditions. The MIC was defined as the lowest concentration of the test sample that completely inhibited visible bacterial growth. MPEO was dissolved in 0.3% (v/v) Tween 80 to ensure uniform distribution. Untreated bacteria were used as the growth control, while Tween 80 and SDS served as negative controls.

### Vapor-phase diffusion assay

Vapor-phase activity was evaluated in triplicate. Briefly, 30 µL of sonicated or non-sonicated MPNE-SDS and MPEO was carefully pipetted onto sterile filter disks and allowed to be fully absorbed. The disks were then placed on the inverted lids of blood agar plates previously inoculated with 100 µL of *H. pylori* suspension (10⁷ CFU/mL). The plates were sealed with parafilm and incubated at 37 °C for 72 h under microaerophilic conditions. Following incubation, the diameter of the inhibition zone (IZ) was measured. Untreated bacteria and Tween 80/SDS served as controls^80^.

### Atomic force microscopy (AFM)

Structural alterations of *H. pylori* (HP-5) were analyzed using an AFM (Dimension Icon, Bruker). Treated and untreated bacterial suspensions with MPNE-SDS were centrifuged, deposited on mica, air-dried, and then imaged^43^. Topographic images were acquired in PeakForce Tapping mode using a silicon cantilever with a nominal spring constant of 0.4 N/m. The images were captured over a scan area of 10 × 10 μm² with a resolution of 512 × 512 pixels and a scan rate of 0.8 Hz.

### Evaluation of whole-cell urease activity

Urease activity was measured using the phenol red method in triplicate^81,82^. *H. pylori* HP-5 (10^7^ CFU/mL) was incubated with MPNE-SDS at concentrations of ½ MIC, MIC, 2×MIC, and 4×MIC in 96-well plates for 72 h under microaerophilic conditions. After adding 100 µL urea medium, phosphate buffer (pH 6.8) containing 100 mM urea and 0.001% phenol red, absorbance was recorded at 630 nm after 50 min. The percentage reduction in urease activity was calculated using the formula:

$$\left[ {\left( {{\text{activity without MPNE}} - {\text{SDS }} - {\text{ activity with MPNE}} - {\mathrm{SDS}}} \right){\text{ }}/{\text{ }}\left( {{\text{activity without MPNE}} - {\mathrm{SDS}}} \right)} \right]{\text{ }} \times {\mathrm{1}}00.$$

Bacterial viability was qualitatively assessed by plating aliquots of treated *H. pylori* suspensions onto blood agar plates following the 72-hour incubation period. Plates were incubated under microaerophilic conditions at 37 °C for 72 h, and the presence of colony formation was recorded as an indicator of viable bacteria. Tween 80 and SDS were included as negative controls. The concentration of MPNE–SDS required to achieve 50% reduction in whole-cell urease activity (IC₅₀) was determined using GraphPad Prism software.

### pH effect on antibacterial activity of MPNE-SDS

MPNE-SDS efficacy was evaluated under acidic (pH 4; 100 mM citrate buffer supplemented with 10 mM urea) and neutral (pH 7; 10 mM phosphate buffer) conditions. MPNE-SDS at its MIC was added to each buffer, followed by inoculation with 100 µL of *H. pylori* HP-5 suspension (10⁷ CFU/mL). The mixtures were incubated at 37 °C with shaking at 150 rpm, and 100 µL aliquots were collected at 0, 15, 30, and 60 min. The pH values were continuously monitored at all time points to ensure stability throughout the experiment. Each aliquot was immediately diluted in fresh Brucella broth supplemented with 10% FBS. After incubation at 37 °C for 3 days, bacterial growth was evaluated by measuring optical density at 600 nm. Sterile medium and untreated bacterial cultures were included as controls, and MPNE-SDS alone (without bacteria) was used as a blank to correct for any background turbidity^25,83,84^.

### Biofilm inhibition and eradication assays

The antibiofilm activity of MPNE-SDS was evaluated in flat-bottom 96-well microtiter plates. For biofilm inhibition assays, *H. pylori* HP-3 suspension (10^6^CFU/mL) was incubated with MPNE-SDS at concentrations ranging from 6.25 to 200 µL/mL at 37 °C for 3–5 days under shaking conditions (120 rpm)^85^. For biofilm eradication assays, 3-day-old pre-formed biofilms were gently washed with PBS to remove non-adherent cells, then treated with the same concentrations of MPNE-SDS for 24 h at 37 °C. Following incubation, biofilms were washed with PBS, and the biofilm biomass was quantified using 0.1% (w/v) crystal violet staining for 15 min at room temperature. After staining, excess dye was removed, and the wells were washed with sterile distilled water. To solubilize the bound crystal violet, 250 µL of 95% ethanol was added to each well and incubated for 20 min. The absorbance was measured at 570 nm using a microplate reader^86^. Untreated biofilms served as growth controls, while wells containing sterile medium without bacteria were used as blank controls. The concentrations of MPNE-SDS required to inhibit 50% of biofilm formation (IC₅₀) or to eradicate 50% of pre-formed biofilms (EC₅₀) were determined from dose–response curves using nonlinear regression. The cutoff optical density (ODc) was set at 0.2, corresponding to the mean OD of the negative control wells under the same experimental conditions. This threshold was used to classify his threshold was used to classify biofilm formation^87^. All experiments were performed in triplicate.

### Biofilm observation by scanning electron microscopy (SEM)

Biofilm morphology under inhibitory conditions was examined using SEM (S-4800, Hitachi, Japan). *H. pylori* HP-3 biofilms were developed on sterile coverslips in the presence or absence of MPNE-SDS at ½ MIC, MIC, and 4 MIC. After incubation, biofilms were gently washed three times with PBS and fixed with 2.5% glutaraldehyde. The fixed samples were dehydrated through a graded ethanol series, air-dried, sputter-coated with gold, and imaged using SEM to assess structural alterations associated with biofilm inhibition^88^.

### Statistical analysis

All experiments were performed in triplicate. Data are expressed as mean ± standard deviation (SD). Statistical analyses were performed using GraphPad Prism 9.5, and statistical significance was set at *p* < 0.05.

## Supplementary Information

Below is the link to the electronic supplementary material.

Supplementary Material 1

## Data Availability

The results from the current study are available from the corresponding author on reasonable request: Attaran B. (attaran.b@gmail.com).
