# Supplementary material for: Multifunctional activity of Mentha piperita essential oil nanoemulsion against Helicobacter pylori
Source: Sci Rep. 2026 May 18;16:22450. doi: 10.1038/s41598-026-52730-1 (PMC13377183; doi:10.1038/s41598-026-52730-1)
Supplement: Supplementary file 1 — Supplementary Material 1 [file 41598_2026_52730_MOESM1_ESM.pdf]

Multifunctional Activity of *Mentha piperita* Essential Oil Nanoemulsion against *Helicobacter pylori*

Golnaz Ebrahimi[1], Bahareh Attaran[1,2]*, Ali Mohammadi[1,2]

[1] Department of Microbiology, Faculty of Biological Sciences, Alzahra University, Tehran, Iran
[2] Research Center for Applied Microbiology and Microbial Biotechnology (CAMB), Alzahra University, Tehran, Iran

*Corresponding author: Bahareh Attaran

Email: attaran.b@gmail.com

Table S1. GC-MS analysis of *Mentha piperita* essential oil (MPEO) showing chemical composition and relative content (%)

No.	Compound	Content (%)
1	α-Pinene	0.55
2	Sabinene	0.16
3	β- Pinene	0.55
4	β- Myrcene	0.1
5	Delta-3 Carene	0.28
6	Cymene	0.11
7	**1,8-Cineole**	**2.54**
8	**Limonene**	**3.07**
9	**Menthone**	**21.17**
10	**Isomenthone**	**2.85**
11	Menthofuran	6.63
12	Isomenthol	2.11
13	**Menthol**	**51.20**
14	p-Menthan-1-0	0.24
15	Levomenthol	0.3
16	**Pulegone**	**2.1**
17	p-Menth-3-ene	0.15
18	**Menthyl acetate**	**4.12**
19	α-Cubebene	0.07
20	α-Ylangene	0.08
21	α-Bourbonene	0.11
22	**Trans-Caryophyllene**	**0.95**
23	Germacrene-D	0.41
24	Germacrene-D	0.07
	Total	99.01

Table S2. Antibiotic susceptibility patterns of clinical *H. pylori* isolates.

Strain	AMX (25 µg)	CLR (15 µg)	LEV (5 µg)	TET (30 µg)	MTZ (5 µg)
HP-1	S	S	S	S	R
HP-2	S	S	S	R	R
HP-3	S	R	R	R	R
HP-4	S	R	R	R	R
HP-5	S	S	R	R	R

AMX: Amoxicillin; CLR: Clarithromycin; LEV: Levofloxacin; TET: Tetracycline. S = sensitive, R = resistant. Susceptibility was determined according to standard disc diffusion methods.

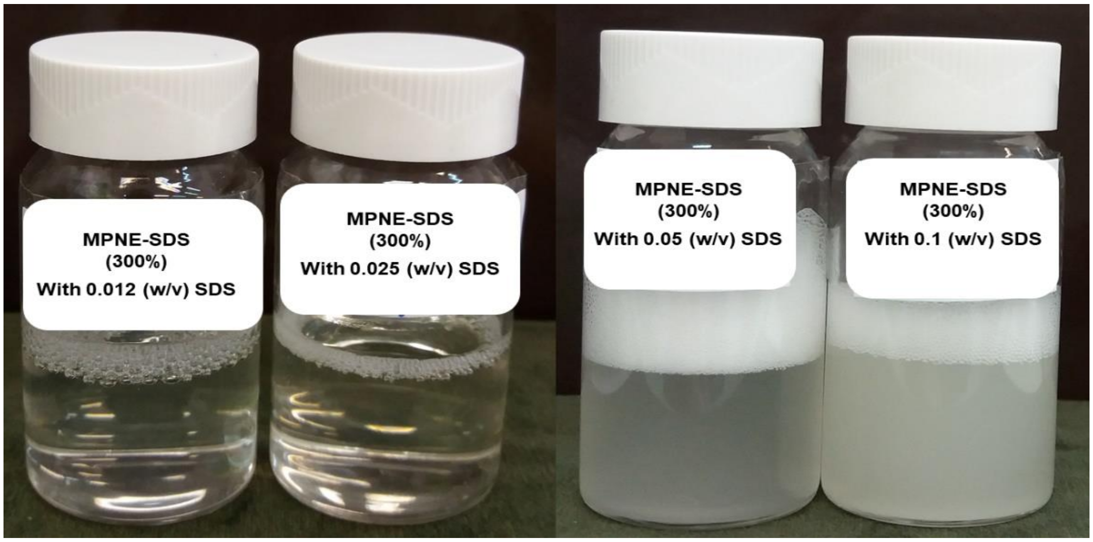

Fig. S1. Visual clarity of MPNE formulations with different SDS concentrations (0.012, 0.025, 0.05, and 0.1 w/v). The 0.012 and 0.025 formulations showed high transparency, while 0.05 and 0.1 exhibited turbidity and foaming.

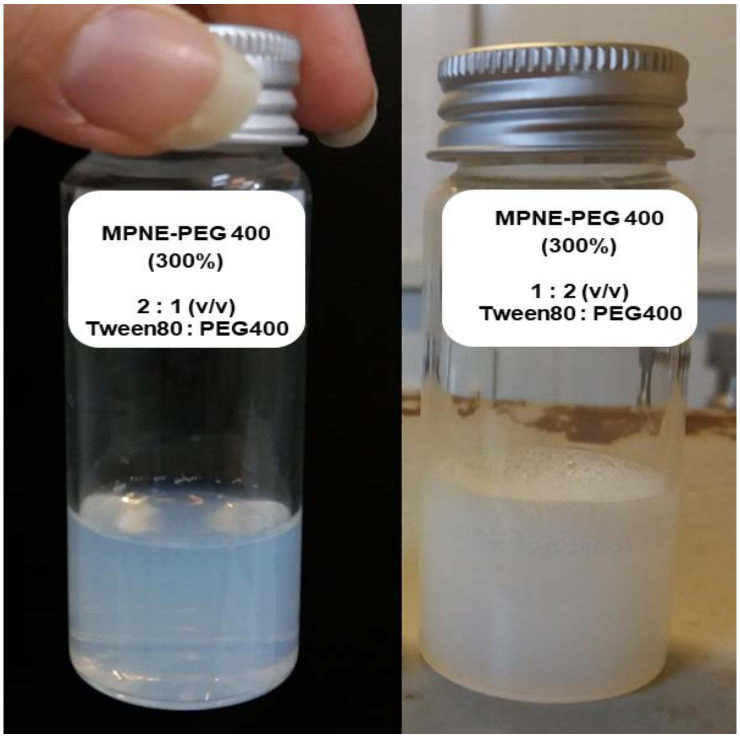

Fig. S2. Visual assessment of MPNE formulations with different Tween 80: PEG 400 ratios (2:1 and 1:2 v/v).
